# Cardiovascular effects of immunosuppression agents

**DOI:** 10.3389/fcvm.2022.981838

**Published:** 2022-09-21

**Authors:** Aly Elezaby, Ryan Dexheimer, Karim Sallam

**Affiliations:** ^1^Stanford Cardiovascular Institute, Stanford University School of Medicine, Stanford, CA, United States; ^2^Division of Cardiovascular Medicine, Department of Medicine, Stanford University, Stanford, CA, United States

**Keywords:** immunosuppression, cardiovascular, hypertrophy, hypertension, mitochondria, fibrosis, toxicity

## Abstract

Immunosuppressive medications are widely used to treat patients with neoplasms, autoimmune conditions and solid organ transplants. Key drug classes, namely calcineurin inhibitors, mammalian target of rapamycin (mTOR) inhibitors, and purine synthesis inhibitors, have direct effects on the structure and function of the heart and vascular system. In the heart, immunosuppressive agents modulate cardiac hypertrophy, mitochondrial function, and arrhythmia risk, while in vasculature, they influence vessel remodeling, circulating lipids, and blood pressure. The aim of this review is to present the preclinical and clinical literature examining the cardiovascular effects of immunosuppressive agents, with a specific focus on cyclosporine, tacrolimus, sirolimus, everolimus, mycophenolate, and azathioprine.

## Introduction

**Figure 1:**
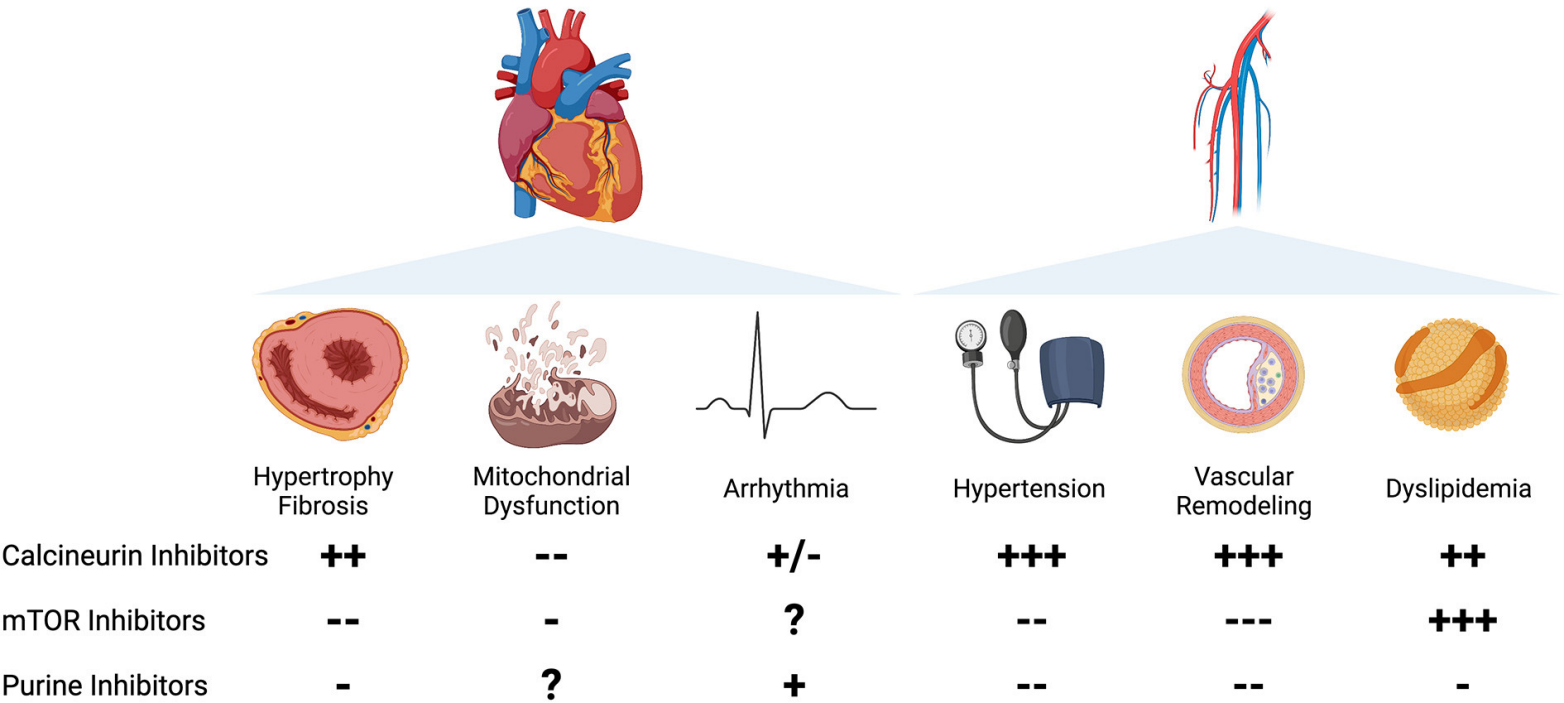
Left Panel: Cardiac effects of immunosuppression. Column A: Calcineurin inhibitors are associated with increased hypertrophy in clinical studies, with mixed preclinical evidence. mTOR inhibitors are associated with a decrease in cardiac hypertrophy in patients and animal studies. Purine synthesis inhibitors prevent cardiac remodeling in limited evidence in preclinical studies. Column B: Calcineurin inhibitors, particularly CsA, prevent mitochondrial dysfunction and mPTP opening. mTOR inhibitors may prevent mitochondrial dysfunction in preclinical studies. The effects of purine synthesis inhibitors on mitochondrial function in the heart are unknown. Column C: Calcineurin inhibitors are associated with arrhythmia in limited clinical case reports, with mixed effects in animal studies. The effects of mTOR inhibitors on arrhythmia are unknown. Purine synthesis inhibitors, particularly azathioprine, are weakly associated with increased atrial arrhythmias in clinical case reports. Right Panel: Vascular effects of immunosuppression. Column A: Hypertension. Calcineurin inhibitors are strongly associated with an increased incidence of hypertension in preclinical and clinical studies. mTOR inhibitors and purine synthesis inhibitors have a vasodilatory effect in animal models and limited clinical studies. Column B: Vascular remodeling. Calcineurin inhibitors are strongly associated with proliferative vasculopathy and vascular inflammation. mTOR inhibitors protect against vascular damage in clinical studies and preclinical models. Purine synthesis inhibitors are associated with improvement in vascular remodeling in preclinical studies and limited clinical reports. Column C: Dyslipidemia. Calcineurin inhibitors are associated with increased total serum cholesterol and LDL. mTOR inhibitors, particularly sirolimus, are strongly associated with an increase in serum cholesterol and triglycerides. Purine synthesis inhibitors are weakly associated with improvement in serum lipids.

Medications that target and downregulate the immune system are utilized for the prevention and treatment of a variety of conditions, including neoplasms, autoimmune diseases, and acute rejection after solid organ transplantation (1). In a recent cohort, 2.8% of the adult population was treated with long-term immunosuppressive medications, consistent with prior self-reported estimates (2, 3). In addition to the well described increased risk of infection and malignancy in chronically immunosuppressed patients, many of these agents exhibit direct effects on the cardiovascular system including risk of left ventricular (LV) hypertrophy, myocardial fibrosis, arrhythmia, hypertension, dyslipidemia, and coronary atherosclerosis (4). Herein, we focus on the cardiovascular effects and mechanistic underpinnings of calcineurin inhibitors (CNI), mammalian target of rapamycin (mTOR) inhibitors, and purine synthesis inhibitors (Figure 1).

## Hypertrophy and fibrosis

Cardiac hypertrophy is a feature of adverse cardiac remodeling that may be driven by genetic or acquired factors. Hypertrophy is frequently seen in association with diastolic dysfunction and represents an important marker for adverse remodeling (5, 6). Much of the focus on immunosuppression-induced cardiac remodeling has been on the effects on cardiac hypertrophy in native or transplanted hearts (7–9) (Table 1).

**Table 1:** Studies examining effects of immunosuppression on cardiac hypertrophy and fibrosis.

**Agent**	**Species**	**Condition**	**Hypertrophy/Fibrosis**	**Studies**
CsA	Rat, mouse	TAC	Attenuated LVH	(10–14)
	Mouse	TAC	No effect	(7, 15, 16)
	Rat	SHR	No effect	(16–18)
	Mouse	Gαq	Attenuated LVH	(19)
	Rat	Cardiac fibroblasts	No effect	(20)
	Rat	Cardiac fibroblasts	Induced fibrosis	(21–23)
	Rat	Langendorff	Decreased scar	(24)
	Human	Transplant	Increased LVH	(25–28)
	Human	LVH, HCM, CAD	Attenuated LVH	(29)
	Human	STEMI	Decreased scar	(30)
	Human	STEMI	No effect	(31–34)
Tacrolimus	Rat	SHR	Attenuated LVH	(35, 36)
	Mouse	Genetic HCM	Exacerbated LVH	(37)
	Rat	SHR, TAC	No effect	(16)
	Human	Transplant	Increased LVH	(26, 27, 38–40)
Sirolimus	Rat	Phenylephrine	Attenuated LVH	(41)
	Mouse, Rat	TAC	Attenuated LVH	(42, 43)
	Rat	Adriamycin	Attenuated fibrosis	(44)
	Mouse	Leprdb diabetic	Prevented fibrosis	(45)
	Rat	Zucker obese	Prevented fibrosis	(46)
		Zucker lean	Increased fibrosis	
	Human	Transplant	Regressed LVH	(47–49)
Everolimus	Human	Transplant	Attenuated LVH, fibrosis	(50, 51)
	Human	Transplant	No effect on LVH	(52–55)
	Rat	Metabolic syndrome	Attenuated LVH, fibrosis	(56)
MMF	Rat	Ischemia-reperfusion	Prevented apoptosis	(57)
	Rat	Myocarditis	Prevented LV dysfunction	(58)

TAC, Transverse Aortic Constriction; SHR, Spontaneously hypertensive rat; LVH, Left ventricular hypertrophy; HCM, hypertrophic cardiomyopathy; CAD, coronary artery disease; STEMI, ST elevation myocardial infarction.

### Calcineurin inhibitors

Calcineurin, a calcium and calmodulin-dependent phosphatase, plays a pivotal role in cardiac hypertrophy by translocating to the nucleus and dephosphorylating NFAT, allowing it to transcribe genes to activate hypertrophy in cardiomyocytes. Cyclosporine (CsA) binds to cyclophilin A, forming a complex with high affinity for calcineurin, which in turn inhibits its nuclear translocation. This is hypothesized to inhibit activation of NFAT-mediated hypertrophy (59). Tacrolimus binds to FK506-binding protein (FKBP12) to inhibit calcineurin activity driving reduced NFAT-mediated transcription of hypertrophic genes.

In early animal experiments, CsA successfully prevented or attenuated cardiac hypertrophy in mice overexpressing contractile elements (10, 29), genetic predispositions to hypertrophy (19), and treatment with exogenous chemical signals promoting hypertrophy (11, 15, 60). However, these data were challenged by the failure of CsA to prevent hypertrophy in several models of hypertension or pressure overload (16, 35, 61). Tacrolimus has also yielded mixed results. In murine models of genetic hypertrophic cardiomyopathy, tacrolimus exacerbated cardiac hypertrophy (37). In animal models of hypertrophy induced by phenylephrine stimulation, spontaneously hypertensive rats, or aortic banding, tacrolimus treatment had variable effects, with exacerbation or amelioration of the hypertrophic phenotype (16, 38, 61, 62).

Some hypothesized that the mixed results were driven by variability in hypertrophic signaling from genetic/sarcomeric-driven hypertrophic signaling vs. adaptive chemical or afterload-driven hypertrophy (37, 59). This hypothesis is somewhat weakened by mixed data for transverse aortic constriction rodent models.

Subsequent investigations suggested that CsA-induced effects on hypertrophic remodeling may be driven by increased fibrosis. Multiple studies have shown that CsA treatment led to increases in MMP2, MMP9, and Collagen I in dose dependent manner (20–22, 63). Rat hearts treated with CsA exhibited increased fibrosis/collagen content (64). Similar data of increased collagen deposition in response to tacrolimus treatment was observed in human induced pluripotent stem cell-derived cardiac organoids treated with tacrolimus (65). The *in vitro* findings suggest that increased fibrosis is not a result of calcineurin-induced hypertension.

Notwithstanding some of the conflicting data in animal models, the data from humans have been fairly consistent as to the effects of CsA and tacrolimus on human hearts. Endomyocardial biopsies from heart or liver transplant patients treated with CsA showed structural distortion, increased fibrosis, and increased collagen levels (25, 26). Furthermore, patients treated with CsA and tacrolimus had hypertrophy or increased LV mass on autopsy or imaging (8, 26, 27, 39, 40). A clinical trial investigating the effect of CsA in patients with hypertrophic cardiomyopathy was initiated, but it is unclear if the study was completed and findings, if any, were not published (66).

Despite some earlier reports of amelioration of cardiac hypertrophy by CNI, there is no clear evidence in humans to corroborate this finding. Supported by *in vitro* and human data, a consistent signal of increased hypertrophy and fibrosis associated with CNI treatment is observed (23, 28). Cellular data highlight that the increase in LV mass may be driven primarily by CNI-induced increase in fibrosis and collagen deposition rather than cardiomyocyte remodeling.

### mTOR inhibitors

mTOR inhibitors, such as sirolimus and everolimus, inhibit mammalian target of rapamycin complex I, thereby inhibiting downstream pathways driving cell growth, proliferation, and survival. There are notable differences between sirolimus and everolimus (67). Everolimus is the 40-O-(2-hydroxyethyl) derivative of sirolimus, and differs in its subcellular distribution, pharmacokinetics and binding affinity. Compared to sirolimus, everolimus has higher bioavailability and shorter half-life. Both drugs form a complex with FKBP-12, which binds mTOR. However, everolimus binding to FKBP-12 is ~3-fold weaker than that of sirolimus, leading to significant differences in inhibition of mTORC2 activation and downstream effects (68, 69). Clinically this has translated into differences in side effect profile and potency of each drug.

This class of drugs has garnered significant interest in solid organ transplantation owing to salutary effects on renal function, allograft vasculopathy and malignancy risk (70). Sirolimus has been shown to reduce cardiac hypertrophy and fibrosis in animal models of pressure overload, uremia, and adriamycin induced cardiomyopathy (42, 43, 71). In a rat model of myocardial infarction, everolimus improved post-infarct remodeling (72) although in the recently published CLEVER-ACS trial of patients with myocardial infarction, there everolimus treatment had no effect on myocardial remodeling (73). Cellular data suggest that attenuation of adverse cardiac remodeling by mTOR inhibitors may be related in part to reduced cardiac fibroblast proliferation and collagen secretion (65).

The favorable signal for sirolimus has been validated in human studies, which largely compared outcomes to subjects treated with CNI. Sirolimus has been associated with improvement in diastolic dysfunction and filling pressures, possibly through attenuation of fibrosis (47–49). In patients with heart transplantation, everolimus treatment was associated with less myocardial fibrosis than mycophenolate treatment by biopsy and imaging (50, 51). The data in kidney transplant patients has been more mixed with some suggesting less LV hypertrophy with the use of everolimus (74), while a number of randomized trials showed no difference in LV mass index after conversion from CsA to everolimus post-kidney transplant (52–55). The incidence of adverse cardiovascular events from these studies was mixed with the majority showing no differences in outcomes (75–77). This discordant signal may be related to the fact that kidney transplant recipients often have concomitant hypertension and activation of the renin-angiotensin system that may have already contributed to significant adverse cardiac remodeling prior to kidney transplant—making it less likely to observe differences following kidney transplantation (75, 78). Additionally, most of the studies may have been underpowered to detect differences in cardiovascular outcomes.

### Purine synthesis inhibitors

Purine synthesis inhibitors block cell proliferation by preventing the synthesis of DNA and RNA during S phase of the cell-cycle. Mycophenolate mofetil (MMF) treatment has been shown to prevent or attenuate ischemic injury and autoimmune myocarditis in animal models, with reduced secretion of inflammatory markers such as TLR4, NFκB, BAX expression, and TNFα (57, 58). There are no human studies suggesting a link between cardiac hypertrophy or fibrosis in association with MMF or azathioprine use.

## Mitochondrial dysfunction

Mitochondria constitute a third of cardiomyocyte volume, and the heart, as a metabolically active organ, relies heavily on mitochondrial ATP production (79). Mitochondrial dysfunction is a feature of multiple types of cardiomyopathy, as it confers oxidative stress and changes in energetics to drive adverse cardiac remodeling. Immunosuppressive agents can exert direct effects on mitochondrial health to modulate cardiac remodeling and this has been subject of much investigation (Table 2).

**Table 2:** Studies examining effects of immunosuppression on cardiac mitochondrial function.

**Agent**	**Species**	**Condition**	**Mito function**	**Studies**
CsA	Rat	Isolated Mito	Protected from Ca^2+^ overload, prevented mPTP opening	(80)
	Rat	Hypothermia	Improved ATP levels	(81)
	Rat	IR injury	Prevented mito injury	(82, 83)
	Mouse	Mito DNA mutations	Prevented mito injury	(84)
	Pig, Rat	Cardioplegic arrest	Prevented mito injury	(85, 86)
	Pig	HFpEF	Attenuated mito dysfunction	(87)
	Mouse	Adriamycin	Prevented loss of mito membrane potential	(88)
	Feline	Endotoxemia	Normalized mito respiration	(89)
Tacrolimus	Mouse	Adriamycin	Did not prevent loss of mito membrane potential	(88)
	Feline	Endotoxemia	Normalized mito respiration	(89)
	Canine, Mouse	IR injury	Prevented loss of mito GSH and attenuated mito dysfunction	(90, 91)
Sirolimus	Mouse	Injection	Inhibited mito respiration	(92)
	Mouse	IR injury	Inhibited apoptosis, opened mito KATP channel	(93)

Mito, Mitochondrial; IR, ischemia-reperfusion; HFpEF, Heart failure with preserved ejection fraction.

### Calcineurin inhibitors

Cyclophilin D is a protein in the inner mitochondrial matrix involved in opening of the mitochondrial permeability transition pore (mPTP) (94). mPTP opening results in mitochondrial calcium overload, release of cytochrome C, a process involved in apoptosis and implicated in myocardial ischemia-reperfusion (IR) injury (80). CsA interacts with cyclophilin D thereby preventing mPTP opening and protecting the mitochondria from calcium overload. Tacrolimus does not bind cyclophilin D and the effects of tacrolimus on mitochondrial function and mPTP opening are less defined. Multiple animal studies have sought to define the effect of both drugs on mitochondrial function.

CsA prevented mitochondrial-mediated injury and improved myocardial recovery in models of hypothermia, IR injury, and inborn errors of mitochondrial DNA polymerase (81–86, 95). In addition, CsA and/or tacrolimus have been associated with a favorable mitochondrial phenotype in the face of adriamycin treatment, hypoxia or endotoxemia (88–90).

Clinical data on the implications of these findings have been scant. In a single study of patients presenting with ST elevation myocardial infarction, CsA treatment decreased myocardial scar burden, which in combination with pre-clinical evidence provided promise for CsA as a “post-conditioning agent” during myocardial infarction (24, 30). However, follow-up studies failed to show any benefit to CsA treatment in regards to LV function, arrhythmia, or mortality (31–34). The discordance suggests that CsA protection from mitochondrial injury is largely a short term or acute benefit. No human studies to date have evaluated the effect of CNI on mitochondrial structure and function in light of associated cardiac remodeling.

### mTOR inhibitors

Sirolimus has been associated with a reduction in respiration and cellular energetics in cardiomyocytes (92). This effect has been attributed to the observation that mTOR may activate AMP-activated protein kinase to regulate cellular bioenergetics (96). In a mouse model of cardiac IR injury, sirolimus inhibited apoptosis and improved cardiac performance *via* interaction with the mitochondrial ATP-sensitive potassium channel (93) and appears to reduce ER stress and cytochrome C release (97). In brain, sirolimus enhances the distribution of CsA into mitochondria, accentuating its effects of decreasing mitochondrial metabolism, whereas everolimus appears to antagonize the effects of CsA in mitochondria to increase energy metabolism (67, 98). At therapeutically relevant concentrations, everolimus, but not sirolimus, distributes into brain mitochondria (99, 100). As cited above clinical studies have suggested a favorable effect for mTOR inhibitors on cardiac remodeling—but data examining mitochondrial function is lacking.

### Purine synthesis inhibitors

There are no reports of direct effects of MMF and Azathioprine on mitochondrial function in cardiomyocytes or heart tissue.

## Arrhythmia

With described effects on myocardial structural remodeling and intracellular ion transporter function, immunosuppressive therapies may modulate the risk of arrhythmia. This poses significant short- and long-term risks, especially in patients with underlying structural heart disease and heart transplant recipients (Table 3).

**Table 3:** Studies examining effects of immunosuppression on arrhythmia.

**Agent**	**Species**	**Condition**	**Arrhythmia**	**Studies**
CsA	Rat	Injection	Sinus tachycardia, QT prolongation	(101)
	Rat	Oxidant stressor	Failed to suppress ventricular arrhythmia	(102)
	Rabbit	Atrial myocyte	Prevented cardiac alternans, decreased AF	(103)
	Canine	Pacing-induced AF	Prevented downregulation of LT Ca^2+^ channel α-1c expression	(104)
	Canine	Chronic AV block	Prevented polymorphic ventricular tachycardia	(105)
	Mouse	Iron overload	Prevented arrhythmia	(106)
	Human	STEMI	No effect	(31–34)
	Human	Transplant	Case reports of increased arrhythmia	(107, 108)
Tacrolimus	Guinea pig	Injection	Dose-dependent QT prolongation	(109, 110)
	Pig, rat	Isolated myocytes	Increased Ca^2+^ transients, prolonged action potential	(111–114)
	Rat	IR injury	Decreased ventricular arrhythmias	(115)
	Human	Transplant	Case reports of arrhythmias	(116–118)
Azathioprine	Human	Transplant	More atrial arrhythmias than MMF	(119)
	Human	Ulcerative colitis, psoriasis	Case reports of atrial fibrillation	(120–123)

STEMI, ST elevation myocardial infarction AF, Atrial fibrillation; IR, Ischemia-reperfusion; AV, atrioventricular.

### Calcineurin inhibitors

Calcineurin affects intracellular calcium transients in cardiomyocytes *via* modulation of the ryanodine receptor and activation of the NFAT pathway, which drives transcriptional changes in proteins regulating intracellular calcium (124). Calcineurin inhibitors in turn can play a role in mediating changes in calcium transients impacting the electrical phenotype of the heart.

Delineating the precise effect of CNI on calcium regulation in human cardiomyocytes has proven elusive. In some models CsA appeared to reduce sarcoplasmic reticulum (SR) calcium release and cytosolic levels of Ca^2+^ (106). However, other models showed that both CsA and tacrolimus result in increased Ca^2+^ release events and an increase in QT prolongation. A possible mechanism of QT prolongation may be an increase in the duration of Ca^2+^ transients due to blockade of Na^2+^/Ca^2+^ exchanger. It is possible that CsA and tacrolimus exert different electrical phenotypes owing to their differential role in mitochondrial Ca^2+^ regulation and mPTP opening. Nonetheless the results in animal models of both drugs have been equally mixed; in some animal models, the cellular phenotypes of CNI appeared to translate to a reduced propensity to arrhythmia (103, 105, 106), but not in other models (101, 102).

Clinically, in case reports, CsA and tacrolimus induced atrial fibrillation and tacrolimus induced QT prolongation and atrial arrhythmias (107, 116). However, neither signal was seen in clinical trials with either drug suggesting that the arrhythmic risk is low (125, 126).

### mTOR inhibitors

There are no published reports of mTOR inhibitors modulating risk of arrhythmias. The recently published CLEVER-ACS trial showed no difference in atrial arrhythmias in patients treated with everolimus after myocardial infarction (73).

### Purine synthesis inhibitors

Azathioprine use is associated with increased incidence of atrial arrhythmias. In a 3-year randomized controlled trial of azathioprine vs. MMF, heart transplant patients treated with azathioprine had a higher rate of atrial arrhythmias than those on MMF (119). The mechanism for this phenomenon is unknown. There are no published reports of MMF modulating arrhythmia risk.

## Hypertension

Hypertension is a well described side effect of immunosuppressive medication use, particularly CNI, and is associated with increased risk of coronary artery disease, cerebrovascular events, renal dysfunction, and adverse cardiovascular remodeling (Table 4).

**Table 4:** Studies examining effects of immunosuppression on hypertension.

**Agent**	**Species**	**Condition**	**Hypertension**	**Studies**
CsA, Tacrolimus	Rat	Injection	Develop HTN prior to LVH	(101)
	Rat	Isolated arteries	Enhanced vasoconstriction, endothelin-1 receptor activation, decrease in eNOS	(127–129)
	Human	Transplant	Increase in HTN after transplant, more in CsA than tacrolimus	(130–132)
Sirolimus	Rat	Mineralocorticoid	Normalized systolic blood pressure	(133)
	Bovine	Endothelial cells	Restored eNOS-mediated vasodilation	(134)
	Human, mouse	PAH	Alleviated hypoxia-induced exacerbation of PAH	(135)
Everolimus	Human	Primary aldosteronism	Associated with improvement in blood pressure	(136)
	Human	Transplant	Lower incidence of HTN compared to CNI	(137)
	Human	PAH	Improvement in pulmonary vascular resistance	(138)
	Human	Renal cell carcinoma	Increased incidence of HTN when used in conjunction with Lenvatinib	(139)
MMF	Mouse	Systemic lupus erythematous	Lowered blood pressure	(140, 141)
	Rat	Lead-induced HTN	Attenuated HTN	(142)
	Rat	Mineralocorticoid HTN	Prevented hypertension	(143, 144)
	Human	Psoriasis, rheumatoid arthritis	Lowered blood pressure	(145)
Azathioprine	Rat	Pregnancy-associated HTN	Attenuated hypertension	(146)
	Human, Rat	PAH	Improved pulmonary vascular resistance	(147)
	Human	Transplant	Less likely to develop hypertension than CsA group	(148)

HTN, Hypertension; PAH, pulmonary arterial hypertension; eNOS, endothelial nitric oxide synthase.

### Calcineurin inhibitors

CNI are known to cause hypertension, with 50–80% of patients reported to have hypertension with chronic use. CsA is associated with a higher incidence compared to tacrolimus (130). CNI are implicated in afferent arteriole vasoconstriction and activation of the renin-angiotensin system, promoting sodium retention and volume expansion (127, 149). Furthermore, CsA and tacrolimus are associated with promoting direct vasoconstriction by one or more of the following mechanisms: increased tone of vascular smooth muscle (128, 150, 151), reduced nitric oxide production (129), and activation of endothelin-1 receptor (129). In cultured murine endothelial and vascular smooth muscle cells, both CsA and tacrolimus were associated with production of proinflammatory cytokines and endothelial activation, with increased superoxide production and NF-kB regulated synthesis of proinflammatory factors, which were prevented by pharmacological inhibition of TLR4. This raises the possibility that a proinflammatory milieu drives chronic endothelial dysfunction, contributing to CNI-induced hypertension (152).

There is some controversy as to whether the clinical hypertrophic phenotype is related to direct myocardial effects or is in fact due an increase in the incidence of hypertension associated with CsA use. Observations that rats treated with CsA develop hypertension prior to myocardial hypertrophy (4, 101, 153–155) supported the notion that perhaps the clinical hypertrophic phenotype is purely related to CNI-induced hypertension rather than direct myocardial effects. While hypertension may be a contributor to the hypertrophic phenotype observed, multiple animal and cellular models have supported a direct effect of CNI on myocardial remodeling.

### mTOR inhibitors

mTOR inhibitors have been associated with a lower risk of hypertension compared to calcineurin inhibitors when used in solid organ transplant recipients (137, 156). The difference between effects of CNI and mTOR inhibitors is likely driven by multiple mechanisms with an overall vasodilatory effect of mTOR inhibitors (157, 158). Sirolimus and everolimus appear to increase nitric oxide production preventing endothelial hyperplasia and dysfunction (133, 134). This promising anti-hypertensive profile has led to the consideration of mTOR inhibitors as a primary therapy for specialized difficult-to-treat populations with hypertension including pulmonary arterial hypertension and primary hyperaldosteronism (138, 139).

### Purine synthesis inhibitors

Purine synthesis inhibitors are not associated with hypertension and may in fact have an antihypertensive effect. In comparison to patients treated with CsA after heart transplantation, those treated with azathioprine were less likely to develop hypertension (148). Lower blood pressures have been reported in patients taking MMF for psoriasis and rheumatoid arthritis (145). Possible mechanisms for the favorable hypertensive profile include: lower pro-inflammatory signaling that drives endothelial dysfunction and hyperplasia, decreased circulating levels of endothelin-1, and reduced sodium reabsorption and neuro-hormonal activation leading to hypertension (142–144). Taken together, these data suggest that purine synthesis inhibitors carry a lower risk of systemic hypertension, and may in fact contribute to favorable mechanisms to reduce hypertension in pulmonary hypertension and renal dysfunction-associated hypertension.

## Vascular remodeling

In addition to effects on hypertension, immunosuppressive agents may directly contribute to abnormal vascular remodeling to drive cardiovascular adverse events, independent of hypertension or dyslipidemia. Defining this risk and the contributing mechanisms for each drug is important in order to ensure appropriate follow up and identify potential actionable targets to modify the risk profile (Table 5).

**Table 5:** Studies examining effects of immunosuppression on vascular remodeling.

**Agent**	**Species**	**Condition**	**Vascular remodeling**	**Studies**
CsA	Mouse	Endothelial and vascular smooth muscle cells	Increased endothelial cell activation, cytokines	(152)
	Rat	Isolated arteries	Increased endothelial dysfunction, oxidative stress, inflammation, smooth muscle proliferation	(128, 159–162)
	Human	Transplant	Associated with proliferative coronary vasculopathy	(163–165)
Tacrolimus	Human Rat	Norepinephrine Acetylcholine	Increased endothelial toxicity, impaired smooth muscle relaxation	(166)
	Human	Transplant	Less vasculopathy than CsA	(167–169)
Sirolimus	Rat	Mineralocorticoid, allografts, shear stress	Inhibited ROS, inflammation, intimal proliferation	(133, 170, 171)
	Pig Rat Human	Smooth muscle	Inhibited cell migration, proliferation	(172–174)
	Human	Transplant	Slowed coronary vasculopathy progression	(175, 176)
	Human	Transplant	Lowered PWV, arterial stiffness	(177, 178)
	Human	Coronary stenting	Prevented intimal proliferation	(179)
Everolimus	Rabbit	Carotid arteries	Improved vascular inflammation, thickening	(180)
	Mouse	LDL-receptor knockout	Prevented atherosclerosis	(181, 182)
	Human	PAH	Improved pulmonary vascular resistance	(138)
	Human	Transplant	Reduced CAV incidence/severity	(183, 184)
	Human	Transplant	No effect on pulse wave velocity	(75)
MMF	Rat	Lead-induced HTN	Decreased inflammation, intimal thickening	(142)
	Human	Transplant	Decrease in atherosclerosis, CAV	(119, 185, 186)
	Human	HUVEC + CNI	Prevented ROS production	(187)
AZA	Rat	Pregnancy-associated HTN	Attenuated endothelial cell dysfunction	(146)
	Rat	Subarachnoid hemorrhage	Attenuated vasospasm, reduced endothelin-1	(188)
	Mouse	Transgenic atherosclerosis	Inhibited atherosclerosis, decreased endothelial monocyte adhesion	(189)
	Human	HUVEC	Decreased cell proliferation	(190)

ROS, reactive oxygen species; PAH, pulmonary arterial hypertension; HUVEC, human umbilical vein endothelial cells; AZA, azathioprine; PWV, pulse wave velocity.

### Calcineurin inhibitors

CNI, particularly tacrolimus, have been associated with increased risk of allograft vasculopathy (167–169, 191). This notable complication of transplanted hearts represents a major driver of graft dysfunction and has significant implications for quality of life and longevity of heart transplant recipients (163–165). This has been replicated in animal models using both tacrolimus and CsA with adverse remodeling features of vascular stiffness, thickening, inflammation and fibrosis noted in treated animals (159, 160). The mechanisms for these include: decreased fibrinolytic activity in vessel walls, increased oxidative stress in endothelial cells, and possibly increased intracellular calcium in vascular smooth muscle cells (161, 162, 192).

### mTOR inhibitors

Both sirolimus and everolimus have been associated with a more favorable vascular profile and their clinical efficacy in reducing the rate of progression of cardiac allograft vasculopathy has led to widespread use in heart transplant recipients (175, 176, 183). In addition to reducing signaling associated with endothelial dysfunction, mTOR inhibitors have been shown to reduce vascular smooth muscle proliferation, intimal hyperplasia, and infiltration by inflammatory cells (170–173, 193, 194). Everolimus, in particular, was shown to reduce pro-inflammatory signaling by decreasing IL-9, VEGF release, and TNFα induced adhesion of endothelial cells (184). These effects have led to wide adoption of everolimus- and sirolimus-eluting stents in the treatment of coronary artery disease (179, 195, 196).

In several trials of kidney transplant patients, a switch from CsA to mTOR inhibitor was associated with stabilization or improvement in parameters of arterial stiffness, including pulse wave velocity (PWV), carotid systolic blood pressure, pulse pressure, and augmentation index (177, 178). One notable exception was a secondary analysis of the ELEVATE trial, where no difference in PWV was found with switch from CsA to everolimus, which was attributed to significant variation in baseline PWV in the study population (75).

In addition to reducing allograft vasculopathy, the anti-vascular proliferation signal conferred by mTOR inhibitors has made the drug class of substantial interest in oncology to suppress tumor neovascularization. Nonetheless, while this anti-proliferation profile offers a substantial benefit, it carries some drawbacks; Namely, both mTOR inhibitor drugs are associated with an increased incidence of lymphedema, which is thought to be driven by inhibition of lymphatic endothelial cell proliferation (197, 198). The incidence of such side effects must be considered in oncologic therapy, where drug dosage is typically higher than that used in transplant immunosuppression (199).

### Purine synthesis inhibitors

Purine synthesis inhibitors appear to confer a beneficial vascular remodeling profile. MMF has been associated with reduced atherosclerosis progression and CAV in patients and animal models (119, 142, 185). Animal models point to a signal of decreased vascular oxidative stress and inflammation as the driving mechanism of that benefit (187, 200, 201). Reduced endothelial and smooth muscle proliferation in association with MMF have also been proposed as a possible mechanism, although the evidence is more limited than for mTOR inhibitors (190).

## Dyslipidemia

Immunosuppressive medications are associated with dyslipidemia. Each drug class is associated with individual variations in affected lipid particles and more importantly in the conferred risk of atherosclerosis (Table 6).

**Table 6:** Studies examining effects of immunosuppression on dyslipidemia.

**Agent**	**Species**	**Condition**	**Dyslipidemia**	**Studies**
CsA	Human	Transplant	Increased total cholesterol, LDL, decreased HDL	(202, 203)
	Human	Transplant	Increased cholesteryl ester transfer protein, lipoprotein lipase activity, decreased lipolysis	(204, 205)
	Human	Transplant	Pro-oxidant effect on LDL	(206, 207)
Tacrolimus	Mouse	High vs low dose	High dose developed hypercholesterolemia, low dose did not	(208)
	Human	Transplant	Less significant increase in LDL, total cholesterol than CsA	(130, 209–213)
	Human	Transplant	Less pro-oxidant effect on LDL than CsA	(206, 207)
	Human Mouse	HUVEC, diabetic mice	Decreases oxidized LDL uptake to endothelial cells, smooth muscle cells	(214–216)
	Mouse	Pcsk9 knockout	Increased PCSK9 expression, leading to decreased LDL receptor expression, increased LDL	(217)
	Human	Transplant	Increase in cholesterol, triglycerides	(70, 218)
	Human	Transplant	Increased apolipoprotein C-III, lipoprotein lipase	(204, 219)
Everolimus	Mouse	LDL-receptor knockout	Increased VLDL/LDL, inhibited atherosclerosis	(181, 182)
	Human	Transplant	No additive increase in total cholesterol and triglycerides	(220)
	Human	Transplant	Similar dyslipidemia to sirolimus	(221)
	Human	Transplant	Decreased oxidized LDL	(222)
	Human	Transplant	No change in lipids, increase in PCSK9	(223, 224)
MMF	Rabbit	High-cholesterol diet	No effect on LDL, HDL, or triglyceride levels	(225)
	Human	Transplant	Cholesteryl ester transfer protein activity unchanged with MMF	(131, 204, 226)
Azathioprine	Human	Transplant	Conversion from CsA decreased total cholesterol, LDL, triglycerides, improved LDL oxidation	(227)
	Human	Transplant	Did not alter serum lipids in comparison to MMF	(228)

### Calcineurin inhibitors

CsA use is associated with a dose-dependent increase in total cholesterol and low-density lipoprotein (LDL) cholesterol, a decrease in high-density lipoprotein (HDL) cholesterol, and an increase in serum triglycerides (202, 203). These changes are driven by a decrease in lipoprotein lipase and an increase in activity of cholesteryl ester transfer protein (204, 229). Additionally, CsA may reduce expression of the LDL receptor, thereby impairing LDL clearance (230–232). Tacrolimus is associated with a similar, but milder, dyslipidemia profile compared to CsA (130, 209–212). CsA appears to be associated with an increase in oxidized LDL, which confers a higher risk of atherosclerosis, while the data for tacrolimus effect on LDL oxidation are mixed (206–208).

### mTOR inhibitors

Sirolimus is a stronger inducer of hyperlipidemia than CNI, associated clinically with an increase in serum LDL and triglyceride levels (70, 218, 233). The mechanism remains unclear, although it may be due to a combination of reduced catabolism, an increase in the free fatty acid pool, increased hepatic production of triglycerides, and secretion of very low density lipoprotein (VLDL) (204, 217). In addition, sirolimus is associated with an increase in serum PCSK9 levels, which acts as a post-transcriptional regulator of LDL receptor expression (234). Clinical data on the risk of dyslipidemia associated with everolimus has been mixed. In clinical studies, everolimus was not associated with an increased risk of dyslipidemia compared to CNI (220, 222, 223, 235–237). However, a meta-analysis comparing mTOR inhibitors to CNI adverse events has noted no difference between sirolimus and everolimus in the incidence of dyslipidemia (238). This suggests that everolimus may contribute to dyslipidemia, but at an intensity that is between CNI and sirolimus.

Interestingly, despite the increase in serum lipids, mTOR inhibitors are associated with an overall lower risk of atherosclerosis (195). Sirolimus reduces oxidized-LDL adhesion and uptake to endothelial cells, and can promote its autophagic degradation (214, 215). Additionally, sirolimus reduces intracellular lipid accumulation in vascular smooth muscle cells, and increases cholesterol efflux *via* increased expression of the ATP binding cassette protein ABCA1 (216). Similarly everolimus treatment in LDL receptor knockout mice, everolimus increased VLDL/LDL levels but reduced the rate of atherosclerosis. Thus, regardless of dyslipidemia profile, mTOR inhibitors appear to result in a net reduction in the rate of atherosclerosis, which may explain the overall clinical benefit observed.

### Purine synthesis inhibitors

Both MMF and azathioprine appear to have a neutral effect on lipids with no significant changes observed in lipid profile in clinical studies (131, 226–228). *In vitro* studies suggest that MMF increases cholesterol efflux, but another study demonstrated inhibition of lipoprotein lipase activity—the opposing effects may explain the net neutral profile conferred by the drug.

## Drug exposure and bioavailability

It is important to note that the bioavailability and exposure levels of the immunosuppression drugs have varied tremendously across clinic and scientific studies in the field. This may explain the differences observed between pre-clinical and clinical studies or even discrepancies between different clinical studies. Part of this variation is not simply investigator mediated, but is driven by variability in clinical practice by geographic area and changes in clinical practice over time. Early CsA trough concentrations in kidney transplant patients ranged 200–500 μg/ml, whereas in Europe, they were typically lower (100–200 μg/ml). Similarly, tacrolimus trough levels ranged 12–20 ηg/ml in the US, and lower in Europe (8–15 ηg/ml). There were also variations in sirolimus and everolimus levels when used in combination with CNI. MMF was previously prescribed at higher doses than is typically used now (2–3 g twice daily to 1 g twice daily) (12, 55, 107, 131).

## Conclusions

Immunosuppressive agents exert significant effects on the heart and vasculature. Mechanistic studies point toward immunosuppression drug-specific influences on changes in cell proliferation, mitochondrial function, inflammatory cytokines, and altered calcium handling as potential mediators of these phenotypes. Calcineurin inhibitors promote cardiac hypertrophy, hypertension, dyslipidemia, and vascular remodeling, while mTOR inhibitors have an anti-proliferative effect with attenuation of cardiac hypertrophy and vascular remodeling despite promoting dyslipidemia. Purine synthesis inhibitor are less well studied, but may have a neutral to mildly positive effect on hypertension and vascular remodeling. These phenotypes are associated with significant morbidity in patients taking immunosuppressive medications, carrying increased risks of heart failure, cardiovascular disease, and kidney dysfunction. While preclinical studies have provided invaluable insight into mechanisms of cardiovascular remodeling, the discordance with clinical data, such as in the case of CNI and hypertrophy, highlights the importance of caution in generalizing the results of cell-based and animal models. Further translational research is needed to identify actionable targets to treat associated cardiovascular side effects of immunosuppression drugs.

## Data availability statement

The original contributions presented in the study are included in the article/supplementary material, further inquiries can be directed to the corresponding author.

## Author contributions

AE, RD, and KS contributed to the writing and figures presented in the manuscript. All authors contributed to the article and approved the submitted version.

## Funding

NIH T32 HL09427411A1 (AE), NIH K08 HL135343 (KS), and American Heart Association and Enduring Hearts Grant # 924127/Sallam and Hollander/2022 (KS).

## Conflict of interest

The authors declare that the research was conducted in the absence of any commercial or financial relationships that could be construed as a potential conflict of interest.

## Publisher's note

All claims expressed in this article are solely those of the authors and do not necessarily represent those of their affiliated organizations, or those of the publisher, the editors and the reviewers. Any product that may be evaluated in this article, or claim that may be made by its manufacturer, is not guaranteed or endorsed by the publisher.
